# Diethyl 3,4-dimethyl­thieno[2,3-*b*]thio­phene-2,5-dicarboxyl­ate

**DOI:** 10.1107/S160053681204593X

**Published:** 2012-11-10

**Authors:** Mehmet Akkurt, Alan R. Kennedy, Sabry H. H. Younes, Shaaban K. Mohamed, Gary J. Miller

**Affiliations:** a Department of Physics, Faculty of Sciences, Erciyes University, 38039 Kayseri, Turkey; b Department of Pure & Applied Chemistry, University of Strathclyde, 295 Cathedral Street, Glasgow G1 1XL, Scotland; c Department of Chemistry, Faculty of Science, Sohag University, 82524 Sohag, Egypt; d Chemistry and Environmental Division, Manchester Metropolitan University, Manchester M1 5GD, England; e Chemistry Department, Faculty of Science, Minia University, 61519 El-Minia, Egypt; f Analytical Sciences, Manchester Metropolitan University, Manchester M1 5GD, England

## Abstract

In the title compound, C_14_H_16_O_4_S_2_, the thieno[2,3-*b*]thio­phene ring systems are planar [maximum deviation = 0.008 (2) Å]. The mol­ecular conformation is stabilized by intra­molecular C—H⋯O hydrogen bonds, while the crystal packing is stabilized by C—H⋯O, C—H⋯π and π–π stacking [centroid–centroid distance = 3.6605 (14) Å] inter­actions, which lead to supra­molecular layers in the *ab* plane.

## Related literature

For the use of thienthio­phenes as versatile precursors for the synthesis of various heterocycles, see: Mabkhot *et al.* (2010 ▶, 2012 ▶); Litvinov (2005 ▶). For their industrial applications, see: Lee & Sotzing (2001 ▶); Heeney *et al.* (2005 ▶); Gather *et al.* (2008 ▶); He *et al.* (2009 ▶). For pharmaceutical values of thieno[2,3-*b*]thio­phenes, see: Jarak *et al.* (2006 ▶); Egbertson *et al.* (1999 ▶). For bond lengths and bond angles in similar compounds, see: Umadevi *et al.* (2009 ▶); Gunasekaran *et al.* (2009 ▶); Wang *et al.* (2008 ▶). For the synthesis of the title compound, see: Comel & Kirsch (2001*a* ▶,*b* ▶). For graph-set descriptions of hydrogen-bond ring motifs, see: Bernstein *et al.* (1995 ▶).
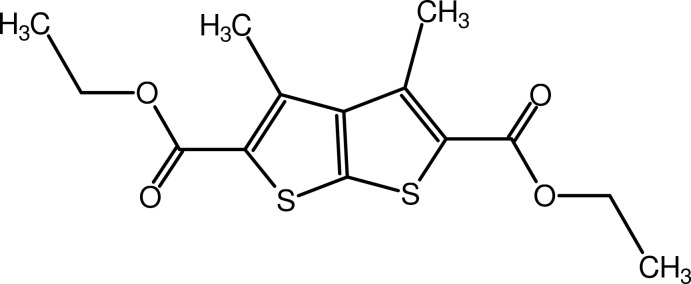


## Experimental

### 

#### Crystal data

C_14_H_16_O_4_S_2_
*M* *_r_* = 312.39
Triclinic, 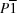

*a* = 7.3497 (3) Å
*b* = 8.4720 (4) Å
*c* = 12.8629 (5) Å
α = 102.770 (3)°
β = 99.545 (3)°
γ = 107.779 (4)°
*V* = 719.96 (6) Å^3^
*Z* = 2
Mo *K*α radiation
μ = 0.38 mm^−1^
*T* = 123 K
0.30 × 0.08 × 0.06 mm

#### Data collection

Oxford Diffraction Xcalibur Eos diffractometer
Absorption correction: multi-scan (*CrysAlis PRO*; Oxford Diffraction, 2010 ▶) *T*_min_ = 0.966, *T*_max_ = 1.000
6901 measured reflections
3486 independent reflections
2661 reflections with *I* > 2σ(*I*)
*R*_int_ = 0.025

#### Refinement

*R*[*F* ^2^ > 2σ(*F* ^2^)] = 0.045
*wR*(*F* ^2^) = 0.106
*S* = 1.04
3486 reflections
185 parameters
H-atom parameters constrained
Δρ_max_ = 0.53 e Å^−3^
Δρ_min_ = −0.34 e Å^−3^

### 

Data collection: *CrysAlis PRO* (Oxford Diffraction, 2010 ▶); cell refinement: *CrysAlis PRO*; data reduction: *CrysAlis PRO*; program(s) used to solve structure: *SHELXS97* (Sheldrick, 2008 ▶); program(s) used to refine structure: *SHELXL97* (Sheldrick, 2008 ▶); molecular graphics: *ORTEP-3 for Windows* (Farrugia, 2012 ▶) and *PLATON* (Spek, 2009 ▶); software used to prepare material for publication: *WinGX* (Farrugia, 2012 ▶) and *PLATON*.

## Supplementary Material

Crystal structure: contains datablock(s) global, I. DOI: 10.1107/S160053681204593X/tk5168sup1.cif

Structure factors: contains datablock(s) I. DOI: 10.1107/S160053681204593X/tk5168Isup2.hkl

Supplementary material file. DOI: 10.1107/S160053681204593X/tk5168Isup3.cml

Additional supplementary materials:  crystallographic information; 3D view; checkCIF report

## Figures and Tables

**Table 1:** Hydrogen-bond geometry (Å, °) *Cg*2 is the centroid of the S2/C1–C4 ring.

*D*—H⋯*A*	*D*—H	H⋯*A*	*D*⋯*A*	*D*—H⋯*A*
C7—H7*A*⋯O1	0.98	2.22	2.980 (3)	133
C8—H8*A*⋯O3	0.98	2.23	2.909 (3)	125
C11—H11*A*⋯O4^i^	0.98	2.53	3.471 (3)	161
C8—H8*C*⋯*Cg*2^ii^	0.98	2.74	3.578 (3)	144

Symmetry codes: (i) 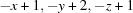; (ii) 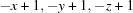.

**Fig. 1.**
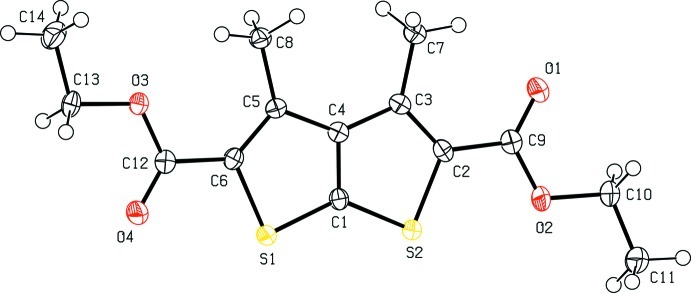
View of the molecular structure of (I) with the atom numbering scheme. Displacement ellipsoids for non-H atoms are drawn at the 50% probability level.

**Fig. 2.**
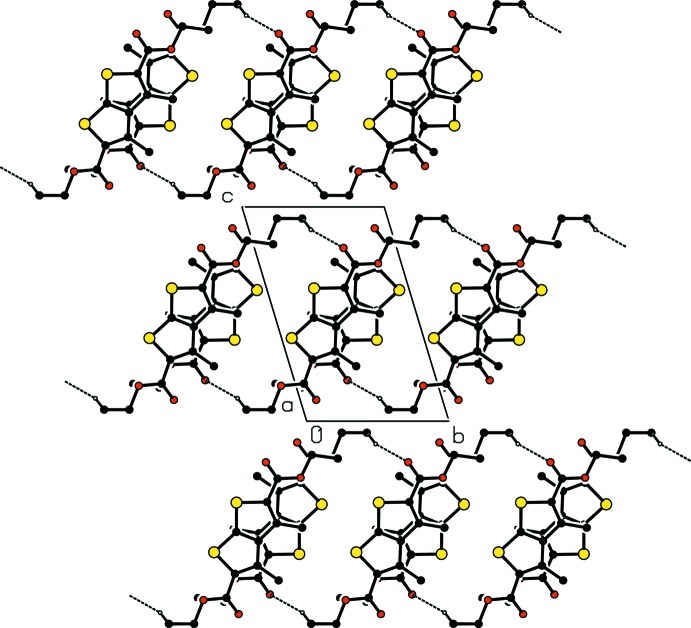
View of the packing and hydrogen bonding of (I) down the a axis. H atoms not involved in hydrogen bonds have been omitted for clarity.

## supplementary crystallographic information

### Comment

Thienothiophene compounds are a great class of sulfur heterocyclic chemistry due their utilities in various applications in industrial and medicinal fields. They have wide variety applications in optical and electronic systems (Gather *et al.*, 2008; He *et al.*, 2009). Besides, thieno[2,3-*b*]thiophenes showed useful reactivities as co-polymerization agents (Lee & Sotzing, 2001) and as semiconductors (Heeney *et al.*, 2005). They have been developed and tested as potential antitumor, antiviral, antiglaucoma drugs, antiproliferation agents, or as inhibitors of platelet aggregation (Jarak *et al.*, 2006; Egbertson *et al.*, 1999). In addition, thienothiophenes have been used as versatile precursors for synthesis of various heterocycles (Mabkhot *et al.*, 2012, Mabkhot *et al.*, 2010; Litvinov, 2005). In view of such important applications, we herein report the crystal structure determination of the title compound (I) to investigate the relationship between its structure and antibacterial activity.

In the title compound, C_14_H_16_O_4_S_2_, the thieno[2,3-*b*]thiophene ring systems are planar with a maximum deviation of 0.008 (2) Å for C2. The values of the bond lengths and bond angles in (I) are in the normal range and comparable to those reported for the similar compounds (Umadevi *et al.*, 2009; Gunasekaran *et al.*, 2009; Wang *et al.*, 2008). The O1–C9–C2–S2, O2–C9–C2–S2, O3–C12–C6–S1 and O4–C12–C6–S1 bond angles are 175.95 (19), -4.8 (3), 176.14 (16) and 4.0 (3)°, respectively.

The intramolecular C7—H7A···O1 and C8—H8A···O3 interactions form six- membered rings, producing *S*(6) ring motif (Table 1; Bernstein *et al.*, 1995). In the crystal, the molecules are linked by intermolecular C—H···O hydrogen bonds (Table 1, Fig. 2), and are further consolidated by C—H···π interactions and π-π stacking [*Cg*1···*Cg*1(-*x*, 1 - *y*, 1 - *z*) = 3.6605 (14) Å; where *Cg*1 is a centroid of the S1/C1/C4–C6 ring] interactions.

### Experimental

The title compound was prepared according to the reported method in literature (Comel & Kirsch, 2001*a*,*b*). Single crystals suitable for X-ray analysis were grown in an ethanol solution of (I) at room temperature over 24 h. *M*.pt: 413 K.

### Refinement

All H atoms were positioned geometrically and refined using a riding model with C—H = 0.98 and 0.99 Å, and with *U*_iso_(H) = 1.2 or 1.5*U*_eq_(C).

### Crystal data

**Table:**

C_14_H_16_O_4_S_2_	*Z* = 2
*M**_r_* = 312.39	*F*(000) = 328
Triclinic, *P*1	*D*_x_ = 1.441 Mg m^−^^3^
Hall symbol: -P 1	Mo *K*α radiation, λ = 0.71073 Å
*a* = 7.3497 (3) Å	Cell parameters from 2806 reflections
*b* = 8.4720 (4) Å	θ = 3.2–29.4°
*c* = 12.8629 (5) Å	µ = 0.38 mm^−^^1^
α = 102.770 (3)°	*T* = 123 K
β = 99.545 (3)°	Rod, colourless
γ = 107.779 (4)°	0.30 × 0.08 × 0.06 mm
*V* = 719.96 (6) Å^3^	

### Data collection

**Table:**

Oxford Diffraction Xcalibur Eos diffractometer	3486 independent reflections
Radiation source: Enhance (Mo) X-ray Source	2661 reflections with *I* > 2σ(*I*)
Graphite monochromator	*R*_int_ = 0.025
Detector resolution: 16.0727 pixels mm^-1^	θ_max_ = 29.5°, θ_min_ = 3.2°
ω scans	*h* = −9→10
Absorption correction: multi-scan (*CrysAlis PRO*; Oxford Diffraction, 2010)	*k* = −11→11
*T*_min_ = 0.966, *T*_max_ = 1.000	*l* = −17→17
6901 measured reflections	

### Refinement

**Table:**

Refinement on *F*^2^	Primary atom site location: structure-invariant direct methods
Least-squares matrix: full	Secondary atom site location: difference Fourier map
*R*[*F*^2^ > 2σ(*F*^2^)] = 0.045	Hydrogen site location: inferred from neighbouring sites
*wR*(*F*^2^) = 0.106	H-atom parameters constrained
*S* = 1.04	*w* = 1/[σ^2^(*F*_o_^2^) + (0.0365*P*)^2^ + 0.4173*P*] where *P* = (*F*_o_^2^ + 2*F*_c_^2^)/3
3486 reflections	(Δ/σ)_max_ = 0.002
185 parameters	Δρ_max_ = 0.53 e Å^−^^3^
0 restraints	Δρ_min_ = −0.34 e Å^−^^3^

### Special details

**Table:**

Geometry. Bond distances, angles *etc*. have been calculated using the rounded fractional coordinates. All su's are estimated from the variances of the (full) variance-covariance matrix. The cell e.s.d.'s are taken into account in the estimation of distances, angles and torsion angles
Refinement. Refinement on *F*^2^ for ALL reflections except those flagged by the user for potential systematic errors. Weighted *R*-factors *wR* and all goodnesses of fit *S* are based on *F*^2^, conventional *R*-factors *R* are based on *F*, with *F* set to zero for negative *F*^2^. The observed criterion of *F*^2^ > σ(*F*^2^) is used only for calculating -*R*-factor-obs *etc*. and is not relevant to the choice of reflections for refinement. *R*-factors based on *F*^2^ are statistically about twice as large as those based on *F*, and *R*-factors based on ALL data will be even larger.

### Fractional atomic coordinates and isotropic or equivalent isotropic displacement parameters (Å^2^)

**Table:**

	*x*	*y*	*z*	*U*_iso_*/*U*_eq_	
S1	0.19179 (8)	0.66605 (7)	0.37995 (4)	0.0197 (2)	
S2	0.42025 (8)	0.92886 (7)	0.61200 (4)	0.0196 (2)	
O1	0.6436 (2)	0.8879 (2)	0.90039 (13)	0.0290 (5)	
O2	0.6126 (2)	1.1054 (2)	0.83495 (12)	0.0258 (5)	
O3	0.0104 (2)	0.1626 (2)	0.26378 (12)	0.0222 (5)	
O4	−0.0238 (2)	0.3735 (2)	0.19133 (13)	0.0273 (5)	
C1	0.3073 (3)	0.7294 (3)	0.51711 (17)	0.0180 (6)	
C2	0.4771 (3)	0.8283 (3)	0.71229 (17)	0.0198 (7)	
C3	0.4109 (3)	0.6525 (3)	0.67430 (17)	0.0179 (6)	
C4	0.3115 (3)	0.5931 (3)	0.55924 (17)	0.0167 (6)	
C5	0.2161 (3)	0.4277 (3)	0.47680 (17)	0.0173 (6)	
C6	0.1453 (3)	0.4496 (3)	0.37738 (18)	0.0187 (6)	
C7	0.4314 (3)	0.5352 (3)	0.74333 (19)	0.0241 (7)	
C8	0.2012 (3)	0.2576 (3)	0.49704 (18)	0.0215 (7)	
C9	0.5860 (3)	0.9392 (3)	0.82492 (18)	0.0208 (7)	
C10	0.7119 (4)	1.2220 (3)	0.94621 (19)	0.0270 (8)	
C11	0.7189 (4)	1.3989 (3)	0.9461 (2)	0.0333 (8)	
C12	0.0360 (3)	0.3267 (3)	0.26830 (18)	0.0200 (7)	
C13	−0.0980 (3)	0.0358 (3)	0.15737 (18)	0.0238 (7)	
C14	−0.1327 (4)	−0.1397 (3)	0.1742 (2)	0.0339 (8)	
H7A	0.51440	0.60450	0.81710	0.0360*	
H7B	0.49260	0.45630	0.70920	0.0360*	
H7C	0.30070	0.46780	0.74890	0.0360*	
H8A	0.09500	0.16430	0.43900	0.0320*	
H8B	0.17240	0.25770	0.56880	0.0320*	
H8C	0.32650	0.23930	0.49660	0.0320*	
H10A	0.84740	1.22180	0.96810	0.0320*	
H10B	0.63870	1.18320	0.99940	0.0320*	
H11A	0.79250	1.43660	0.89370	0.0500*	
H11B	0.78470	1.47890	1.02010	0.0500*	
H11C	0.58420	1.39800	0.92450	0.0500*	
H13A	−0.02020	0.05130	0.10210	0.0290*	
H13B	−0.22530	0.04930	0.13110	0.0290*	
H14A	−0.00580	−0.14950	0.20290	0.0510*	
H14B	−0.20110	−0.22920	0.10380	0.0510*	
H14C	−0.21400	−0.15490	0.22700	0.0510*	

### Atomic displacement parameters (Å^2^)

**Table:**

	*U*^11^	*U*^22^	*U*^33^	*U*^12^	*U*^13^	*U*^23^
S1	0.0227 (3)	0.0181 (3)	0.0164 (3)	0.0062 (2)	0.0022 (2)	0.0049 (2)
S2	0.0231 (3)	0.0171 (3)	0.0170 (3)	0.0065 (2)	0.0030 (2)	0.0041 (2)
O1	0.0353 (9)	0.0283 (10)	0.0183 (8)	0.0094 (8)	−0.0007 (7)	0.0051 (7)
O2	0.0300 (9)	0.0246 (10)	0.0164 (8)	0.0088 (7)	−0.0017 (7)	0.0003 (7)
O3	0.0253 (8)	0.0173 (9)	0.0181 (8)	0.0045 (7)	0.0002 (6)	0.0020 (6)
O4	0.0327 (9)	0.0233 (10)	0.0199 (8)	0.0064 (7)	−0.0002 (7)	0.0045 (7)
C1	0.0162 (10)	0.0191 (12)	0.0167 (10)	0.0050 (9)	0.0030 (8)	0.0037 (9)
C2	0.0196 (11)	0.0256 (13)	0.0152 (10)	0.0092 (9)	0.0043 (8)	0.0063 (9)
C3	0.0156 (10)	0.0218 (12)	0.0186 (11)	0.0074 (9)	0.0055 (8)	0.0083 (9)
C4	0.0130 (10)	0.0188 (12)	0.0187 (10)	0.0050 (8)	0.0053 (8)	0.0063 (9)
C5	0.0157 (10)	0.0173 (12)	0.0201 (11)	0.0065 (9)	0.0059 (8)	0.0061 (9)
C6	0.0171 (10)	0.0175 (12)	0.0199 (11)	0.0054 (9)	0.0048 (8)	0.0033 (9)
C7	0.0279 (12)	0.0230 (13)	0.0210 (11)	0.0095 (10)	0.0029 (9)	0.0074 (10)
C8	0.0225 (11)	0.0188 (12)	0.0218 (11)	0.0063 (9)	0.0035 (9)	0.0063 (9)
C9	0.0164 (11)	0.0251 (13)	0.0203 (11)	0.0071 (9)	0.0053 (9)	0.0052 (9)
C10	0.0321 (13)	0.0239 (14)	0.0191 (12)	0.0081 (10)	0.0013 (10)	0.0010 (10)
C11	0.0400 (15)	0.0257 (15)	0.0266 (13)	0.0098 (12)	−0.0010 (11)	0.0023 (11)
C12	0.0172 (11)	0.0207 (12)	0.0200 (11)	0.0046 (9)	0.0058 (8)	0.0041 (9)
C13	0.0221 (11)	0.0187 (13)	0.0211 (11)	0.0023 (9)	0.0009 (9)	−0.0025 (9)
C14	0.0379 (15)	0.0217 (14)	0.0345 (15)	0.0076 (11)	0.0043 (11)	0.0012 (11)

### Geometric parameters (Å, º)

**Table:**

S1—C1	1.711 (2)	C10—C11	1.484 (4)
S1—C6	1.751 (3)	C13—C14	1.501 (4)
S2—C1	1.712 (2)	C7—H7A	0.9800
S2—C2	1.758 (2)	C7—H7B	0.9800
O1—C9	1.214 (3)	C7—H7C	0.9800
O2—C9	1.334 (3)	C8—H8A	0.9800
O2—C10	1.465 (3)	C8—H8B	0.9800
O3—C12	1.331 (3)	C8—H8C	0.9800
O3—C13	1.459 (3)	C10—H10A	0.9900
O4—C12	1.211 (3)	C10—H10B	0.9900
C1—C4	1.386 (3)	C11—H11A	0.9800
C2—C3	1.360 (3)	C11—H11B	0.9800
C2—C9	1.475 (3)	C11—H11C	0.9800
C3—C4	1.437 (3)	C13—H13A	0.9900
C3—C7	1.495 (3)	C13—H13B	0.9900
C4—C5	1.441 (3)	C14—H14A	0.9800
C5—C6	1.373 (3)	C14—H14B	0.9800
C5—C8	1.495 (4)	C14—H14C	0.9800
C6—C12	1.472 (3)		
			
C1—S1—C6	89.57 (11)	C3—C7—H7C	109.00
C1—S2—C2	89.39 (11)	H7A—C7—H7B	109.00
C9—O2—C10	114.48 (17)	H7A—C7—H7C	109.00
C12—O3—C13	115.57 (17)	H7B—C7—H7C	109.00
S1—C1—S2	132.34 (15)	C5—C8—H8A	109.00
S1—C1—C4	113.81 (17)	C5—C8—H8B	109.00
S2—C1—C4	113.85 (16)	C5—C8—H8C	109.00
S2—C2—C3	113.92 (16)	H8A—C8—H8B	110.00
S2—C2—C9	118.18 (18)	H8A—C8—H8C	109.00
C3—C2—C9	127.9 (2)	H8B—C8—H8C	109.00
C2—C3—C4	111.0 (2)	O2—C10—H10A	110.00
C2—C3—C7	124.9 (2)	O2—C10—H10B	110.00
C4—C3—C7	124.1 (2)	C11—C10—H10A	110.00
C1—C4—C3	111.8 (2)	C11—C10—H10B	110.00
C1—C4—C5	112.16 (19)	H10A—C10—H10B	108.00
C3—C4—C5	136.0 (2)	C10—C11—H11A	109.00
C4—C5—C6	110.3 (2)	C10—C11—H11B	109.00
C4—C5—C8	124.45 (19)	C10—C11—H11C	109.00
C6—C5—C8	125.3 (2)	H11A—C11—H11B	109.00
S1—C6—C5	114.18 (18)	H11A—C11—H11C	109.00
S1—C6—C12	113.10 (17)	H11B—C11—H11C	109.00
C5—C6—C12	132.7 (2)	O3—C13—H13A	110.00
O1—C9—O2	123.4 (2)	O3—C13—H13B	110.00
O1—C9—C2	125.1 (2)	C14—C13—H13A	110.00
O2—C9—C2	111.59 (19)	C14—C13—H13B	110.00
O2—C10—C11	108.25 (19)	H13A—C13—H13B	109.00
O3—C12—O4	124.3 (2)	C13—C14—H14A	109.00
O3—C12—C6	113.56 (19)	C13—C14—H14B	109.00
O4—C12—C6	122.1 (2)	C13—C14—H14C	109.00
O3—C13—C14	106.76 (18)	H14A—C14—H14B	110.00
C3—C7—H7A	109.00	H14A—C14—H14C	109.00
C3—C7—H7B	109.00	H14B—C14—H14C	109.00
			
C6—S1—C1—S2	179.7 (2)	C9—C2—C3—C7	2.3 (4)
C6—S1—C1—C4	0.3 (2)	S2—C2—C9—O1	−175.95 (19)
C1—S1—C6—C5	−0.3 (2)	S2—C2—C9—O2	4.8 (3)
C1—S1—C6—C12	179.25 (18)	C3—C2—C9—O1	4.7 (4)
C2—S2—C1—S1	−179.2 (2)	C3—C2—C9—O2	−174.6 (2)
C2—S2—C1—C4	0.3 (2)	C2—C3—C4—C1	−0.5 (3)
C1—S2—C2—C3	−0.6 (2)	C2—C3—C4—C5	179.2 (3)
C1—S2—C2—C9	180.0 (2)	C7—C3—C4—C1	177.4 (2)
C10—O2—C9—O1	−2.0 (3)	C7—C3—C4—C5	−3.0 (4)
C10—O2—C9—C2	177.3 (2)	C1—C4—C5—C6	−0.1 (3)
C9—O2—C10—C11	−176.3 (2)	C1—C4—C5—C8	178.6 (2)
C13—O3—C12—O4	−0.1 (3)	C3—C4—C5—C6	−179.8 (3)
C13—O3—C12—C6	179.74 (19)	C3—C4—C5—C8	−1.0 (4)
C12—O3—C13—C14	−172.8 (2)	C4—C5—C6—S1	0.3 (3)
S1—C1—C4—C3	179.62 (17)	C4—C5—C6—C12	−179.2 (2)
S1—C1—C4—C5	−0.1 (3)	C8—C5—C6—S1	−178.41 (19)
S2—C1—C4—C3	0.1 (3)	C8—C5—C6—C12	2.1 (4)
S2—C1—C4—C5	−179.66 (17)	S1—C6—C12—O3	176.14 (16)
S2—C2—C3—C4	0.7 (3)	S1—C6—C12—O4	−4.0 (3)
S2—C2—C3—C7	−177.17 (19)	C5—C6—C12—O3	−4.4 (4)
C9—C2—C3—C4	−179.9 (2)	C5—C6—C12—O4	175.5 (3)

### Hydrogen-bond geometry (Å, º)

Cg2 is the centroid of the S2/C1–C4 ring.

**Table:**

*D*—H···*A*	*D*—H	H···*A*	*D*···*A*	*D*—H···*A*
C7—H7*A*···O1	0.98	2.22	2.980 (3)	133
C8—H8*A*···O3	0.98	2.23	2.909 (3)	125
C11—H11*A*···O4^i^	0.98	2.53	3.471 (3)	161
C8—H8*C*···*Cg*2^ii^	0.98	2.74	3.578 (3)	144

Symmetry codes: (i) −*x*+1, −*y*+2, −*z*+1; (ii) −*x*+1, −*y*+1, −*z*+1.
